# Design Principles for EMAT Coils Based on Lorentz Force

**DOI:** 10.3390/s26123624

**Published:** 2026-06-06

**Authors:** Jhon Padilla, Daniel Bernal, Mauricio Barrios Castellanos, Miguel Rios, Juan Argüello, Juan Mantilla, Luis Angel

**Affiliations:** 1 Corporación para la Investigación de la Corrosión, Piedecuesta 681011, Colombia; dbernal@corrosioncic.com; 2 Universidad Pontificia Bolivariana, Medellin 050031, Colombia; mauricio.bc.89@gmail.com (M.B.C.); juan.arguello@upb.edu.co (J.A.); juan.mantilla@upb.edu.co (J.M.); luis.angel@upb.edu.co (L.A.); 3 Fundación Universitaria de San Gil, San Gil 684031, Colombia; miguelrios120@unisangil.edu.co

**Keywords:** NDT, EMAT, ultrasound

## Abstract

EMAT technology for Non Destructive testing is an important method for materials testing in several industries. In EMAT tools, a key issue is the EMAT coils design and implementation. Depending on the type of inspection, the coil type should be selected, and then, its dimensions should be calculated. This paper describes a methodology to select, design and implement EMAT coils based on Lorentz Force for applications such as thickness measurement and crack detection. Unlike previous works that focus on a single coil topology, this study integrates coil selection, dimensional design, COMSOL-based radiation-pattern simulation and experimental validation within a single workflow. Four Lorentz-force coil designs are covered: PCB spiral (CSPCB), 3D-printed spiral (CS3D), PCB meander-line (CMPCB) and 3D-printed meander-line (CM3D). Key design parameters are explicitly addressed: number of turns *N*, outer and inner radii *R* and $r_{0}$, track width *w* and spacing *s* for spiral coils, and meander length and inter-trace distance for meander-line coils. Simulation verification is performed in COMSOL Multiphysics by evaluating the von Mises stress along a semicircular path around the coil to obtain the angular radiation pattern. Experimentally, polar radiation patterns are measured at 500 kHz, 1.9 MHz and 4 MHz on a steel specimen, matching the simulation frequencies, with maximum amplitudes of 32.2, 46.4, 47.9 and 10.6 mV for CSPCB, CS3D, CMPCB and CM3D, respectively, showing consistent agreement between simulated and measured lobe shape and directivity. This work also uses an analogy with radio frequency antennas to better understand the operation of coils through the concept of radiation patterns, in this case in solid materials such as steel.

## 1. Introduction

Electromagnetic Acoustic Transducer (EMAT) technology is a relevant method in Non-Destructive Testing (NDT) across several industries. An EMAT consists of magnets and coils positioned close to the material under inspection; an alternating current applied to the coils gives rise to three physical phenomena: eddy currents, magnetostrictive forces and magnetization. Depending on the geometric arrangement of the magnets and the coils, EMAT coils are classified into two groups, those based on the Lorentz force and those based on magnetostriction. This paper focuses on Lorentz-force-based coils, used in applications such as thickness measurement and crack detection. The coils are fabricated on printed circuit boards (PCB) and using 3D-printing technology.

Although documents describing different types of EMAT coils can be found in the scientific literature, it is difficult to find an article that summarizes in a single document a procedure that allows the design of several types of EMAT coils based on Lorentz Force, including key equations for sizing of different coil types in thickness measurement and crack detection applications. In addition, to better understand the operation of EMAT coils, an analogy with radio-frequency antennas is used: the concept of radiation pattern, well known for antennas, is also useful for the EMAT coils case. Beyond the methodological survey, a central contribution of this work is the fabrication of four physical coils built with two different construction technologies, PCB (CSPCB, CMPCB) and 3D-printing (CS3D, CM3D), and the side-by-side comparison of their COMSOL-simulated and experimentally measured radiation patterns at matched frequencies. This experimental and comparative aspect, rather than a comprehensive literature review, constitutes the principal practical value of the paper.

The paper also reports several simulations and laboratory experiments performed to measure the radiation pattern of each coil. The measurements use a custom test bench that records the amplitude of the received signal over 180° around a transmitter equipped with EMAT coils of different geometries.

Section 2 describes related works about EMAT coils design. Section 3 reviews the methodology for selecting, designing and testing the coils. Section 4 introduces the fundamentals of ultrasound. Section 5 lists the applications of the different wave modes. Section 6 details the design of spiral and meander-line coils. Section 7 reports the radiation-pattern simulations for each coil type, while Section 8 and Section 9 present the experimental setup and the measurement results. The conclusions close the paper.

## 2. Related Work

Several papers describe specific cases involving various types of EMAT coils. In [1], authors describe applications for several types of EMAT coils. Also, in [2], authors explain the types of coils according to the predominant physical phenomenon (Lorentz force or magnetostriction), and explain how to design meander-line-type coils. In addition, in [3], authors describe different types of coils, the underlying physics that explains the EMAT phenomenon, and different applications for each type of coil.

The following table provides a comparison of the sources based on coil geometries, physical phenomena, research methods, and the presence of structured methodologies such as algorithms or flowcharts.

Table 1 lists works that describe different types of EMAT coils, the physical phenomena involved and the methods used for their analysis. The representative documents from that table are described below.

Ogi and Hirao’s book in [3] is one of the most comprehensive documents, describing a large number of coil geometries and presenting various simulations, measurements, and calculations. It even includes different algorithms for diffraction correction and inverse calculation of elastic constants. However, this document does not specifically and concisely describe the steps to perform for the design of the coils in the form of an algorithm or diagram showing the sequence to follow.

Xie’s dissertation in [4], for its part, focuses on meander-line coils that harness the Lorentz force. It also presents an interesting analysis using the Finite Difference Time Domain (FDTD) technique, typically used for antennas, but in this case to analyze the radiation patterns of the coils when generating ultrasound. With respect to the algorithms, the steps proposed by Xie are to couple the electromagnetic models with the ultrasound models, but not as a general methodology for coil design.

**Table 1:** Comparison of several papers describing EMAT coil designs.

Source	Coil Geometries	Predominant Phenomenon	Methods Used	Algorithm or Flowchart
JSNDI (Dobmann) [2]	Meander, Spiral, and toroidal yoke.	Lorentz force and Magnetostriction.	Theoretical principles and industrial applications.	**No** (Explains principles textually).
Hirao & Ogi (EMAT book) [3]	Meander-line, spiral elongated, solenoid, pancake, and Chirp.	Lorentz, Magnetostriction, and Magnetization force.	2D FEM, experimental (EMAR), and inverse calculation.	**Yes** (Iterative diffraction correction).
Caswell (Dissertation) [5]	Archimedean spiral, star, square, and zigzag.	Electromagnetic (antenna context)	NEC4 simulation and radiation pattern measurements.	**Yes** (Genetic Algorithm for optimization).
Pipes Review (Memon & Alhems) [1]	Meander, racetrack, butterfly, and bobbin.	Lorentz and Magnetostriction.	Review of FEM and dispersed experimental data.	**Yes** (Peak detection algorithms).
Xie (Modelling techniques) [6]	Meander, variable-length (VLMLC), and multidirectional.	Lorentz Force.	Hybrid modeling (Analytical EM + FDTD US).	**Yes** (Detailed coupling steps).
Line-focusing (Ogi et al.) [7]	Meander with non-uniform spacing (line focus).	Lorentz and Magnetostriction.	Numerical amplitude simulation and slit defect tests.	**Yes** (Geometric path equations).
Directivity (Xie et al.) [4]	Meander-line coil.	Lorentz Force.	Coupled analytical method (EM and UT).	**Yes** (Transformation methodology).
Rayleigh Wave (Thring et al.) [8]	Linear, meander, and racetrack.	Lorentz Force.	FEA (PZFlex), analytical models, and measurements.	**No** (Presents logical steps textually).
Spiral Coils (Lu et al.) [9]	Spiral coil (pancake).	Lorentz Force.	Circuit-field coupled FEM analysis.	**Yes** (7-step design flowchart).

On the other hand, ref. [5] conducted a study on spiral patch antennas, performing simulations with NEC4. However, his findings are applicable to spiral coils, and the radiation pattern for the ultrasonic field is similar in shape to the radiation pattern used in the antennas, as obtained in our simulations with Comsol and in the measurements performed in our work. The equations for sizing spiral antennas are applicable to spiral coils, but with changes in the frequency range due to the different characteristics of the propagation medium in each case.

The main objective of [7] is the development of a Line-Focusing Electromagnetic Acoustic Transducer (LF-EMAT) capable of exciting and concentrating shear vertical (SV) elastic waves onto a focal line with sharp directivity. The study aims to demonstrate the feasibility of these sensors for detecting very shallow slit-type defects in steel blocks and evaluating their tolerance to liftoff variations. This work describes the theoretical equations that allow the design of meander-line coils. Numerical simulations were also performed to predict the behavior of the meander-line coils. Furthermore, coils were fabricated using the printed circuit board method, and finally, measurements of the reflected signal amplitude were taken to demonstrate that the presence of a crack can be determined based on the amplitude. Nevertheless, the paper work only with meander-line coils and does not present a specific methodology described by means of steps or flowcharts to design the coils, but rather describes the design process across their sections.

The target of [2] is to present a summary of the research and development results obtained by Hans-Juergen Salzburger and his team regarding electromagnetic acoustic transducers (EMAT) and ultrasonic non-destructive testing (UT-NDT). The document aims to highlight the advantages of EMAT application in warm and hot environments, while also addressing its disadvantages and restrictions, as well as the characterization of fatigue damage in austenitic stainless steel at elevated temperatures (300 °C). Also, the fundamental physical mechanisms are described: the Lorentz force (used in conductors) and the magnetostriction effect (used in ferromagnetic materials). To achieve results under hot conditions, the methodology includes designing coils with heat-resistant electrical isolation, external cooling systems (water or gas), and using specific materials for coil carriers, such as ceramic or polyimide. The document describe also some equations for meander-line coil design. However, the document does not present a specific methodology described by means of steps or flowcharts to design the coils.

Finally, the main objective of [9] is to improve the chirp pulse compression technology (PCT) effect in spiral coil electromagnetic acoustic transducers (EMATs). The authors seek to resolve the problem of signal distortion and frequency spectrum loss that occurs when the EMAT system’s frequency response is inconsistent with the chirp signal bandwidth, which typically leads to a decrease in signal-to-noise ratio (SNR) and range resolution. To achieve this, the study focuses on determining the optimal combination of the coil wire diameter and the impedance matching method. To do this, the authors define an algorithm for the design and testing of the coils:

- Define parameters: Establish coil dimensions (wire diameter (d), coil radius (r)).
- Calculate frequency-domain impedance.
- Determine matching: Calculate the parameters of the matching components to maximize power transfer at the central frequency.
- Calculate transient current: Obtain the transient excitation current i(t) from the circuit model.
- Simulate wave generation: Calculate the transient eddy currents, static magnetic field, Lorentz forces, and resulting ultrasonic propagation.
- Capture signal: Obtain the transient ultrasonic signal x(t) using a line integral of the ultrasonic displacement in the receiving area.
- Pulse compression: Perform the convolution of the captured signal with the reference excitation signal to evaluate the peak and width of the main lobe.

As can be seen on this algorithm, there is good approach to a methodology for coil design, but it is focused only on the design of spiral coils, and the signal issues that optimize the main lobe amplitude, but not on the design of different types of coils for different applications such as crack detection and thickness measurement.

As it can be observed in Table 1, although several papers propose algorithms for some parts of the coil design process, it is difficult to find a paper that presents a specific methodology with clear steps for selecting the appropriate coil type for a given application, calculating its dimensions, performing radiation pattern simulations, and conducting validation measurements—all within the same document. The present work fills this gap by integrating, in a single framework, (i) the selection of a Lorentz-force coil topology according to the target ultrasound wave mode and application, (ii) the dimensional design equations for spiral and meander-line coils, (iii) a COMSOL Multiphysics simulation of the angular radiation pattern using von Mises stress on a semicircular path, and (iv) the experimental validation of the radiation pattern at matched frequencies. Therefore, we propose a methodology to assist EMAT tool designers in deciding which coil to use for a given application, how to size it and how to test it.

## 3. Methodology for EMAT Coils Design

The range of EMAT coil types is wide. This paper focuses on Lorentz-force-based coils, given their ease of construction and practicality.

**Figure 1:**
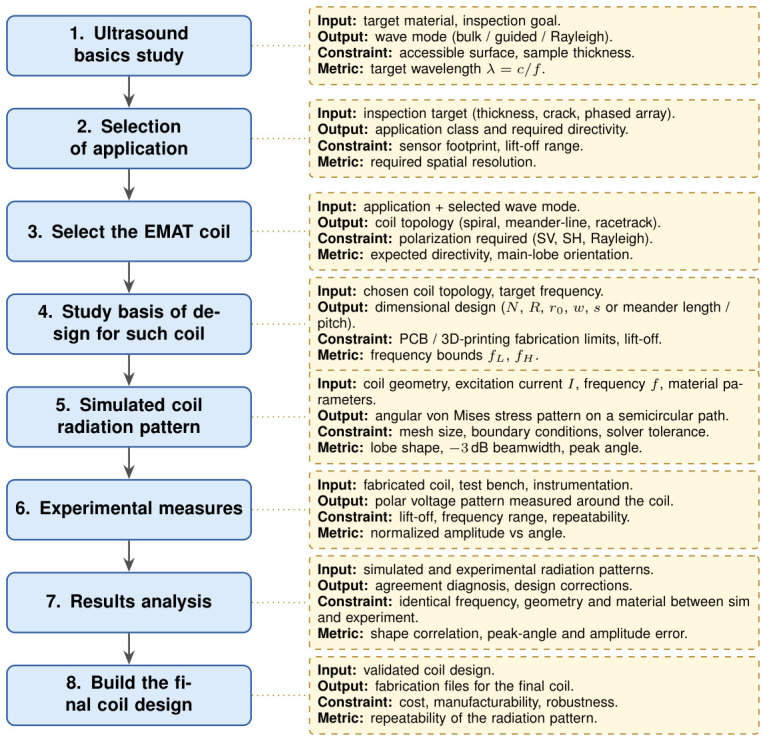
Methodological approach for EMAT coils design. Each of the eight stages is annotated with its inputs, outputs, design constraints and evaluation metrics.

Figure 1 shows the proposed methodology for the development of EMAT coils. The first step is to understand the basic concepts of ultrasound. The second step is to determine the type of application required. The third step is to select the type of EMAT array along with the coil shape. Next, the basic design principles for that coil type should be studied. Then, simulations of the selected coil and EMAT array should be performed, followed by experimental measurements of the radiation pattern, and finally, the results obtained should be analyzed. Each step of this design process will be explained in next sections of this document.

For each step, Figure 1 explicitly lists the expected *inputs* (such as target material, application or coil geometry), the corresponding *outputs* (wave mode, coil topology, dimensional parameters, simulated and measured radiation patterns, and the final fabrication-ready design), the relevant *design constraints* (accessible surface, fabrication limits, mesh and boundary conditions, lift-off, repeatability) and the *evaluation metrics* used to judge each stage (target wavelength, frequency bounds $f_{L}$ and $f_{H}$, −3 dB beamwidth, peak-angle and amplitude error between simulation and experiment). This structured representation turns the original sequential workflow into a domain-specific design framework rather than a generic procedure.

In next sections, the steps of this methodology are explained in detail, starting with the basics of ultrasound, followed by the application selection, coil design, simulation and experimental validation.

## 4. Basics of Ultrasound

**Figure 2:**
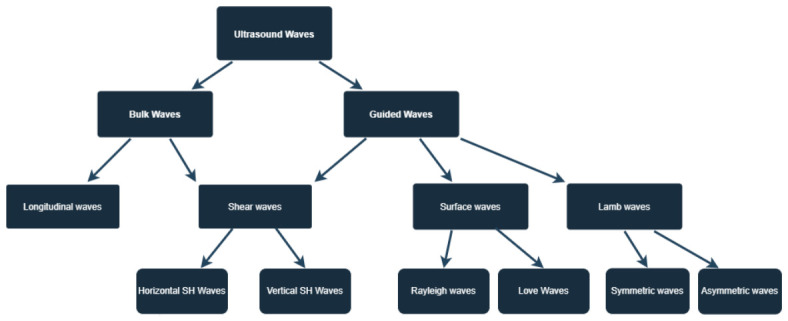
Ultrasound wave categories. Adapted from [1].

Some basics of ultrasonic waves, their applications and how they can be generated using EMAT are reviewed first. In solid materials, ultrasound waves propagate in different modes and are classified into two groups: bulk waves and guided waves. A classification of ultrasound wave types [1] is shown in Figure 2.

**Figure 3:**
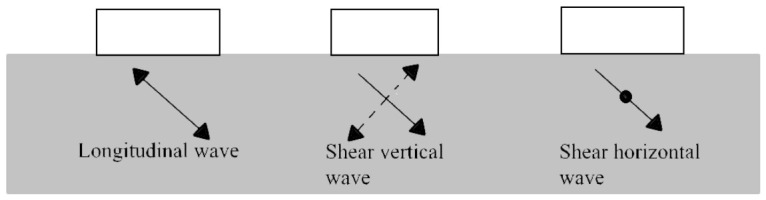
Polarization of ultrasonic bulk waves. Longitudinal (P) waves have particle motion parallel to the propagation direction, while shear vertical (SV) and shear horizontal (SH) waves have particle motion perpendicular to the propagation direction, in the vertical and horizontal planes respectively. Adapted from [2].

Bulk waves are also called free waves because they do not take into account special boundary conditions at the surfaces and interfaces of the test objects. These boundary conditions are relevant for the generation and propagation of guided waves, especially in geometries that act as waveguides. In addition, Bulk waves are classified according to their polarization; there are three possible polarizations [2], which are observed in Figure 3. Polarization types are described as follows:

- Compressional Wave: Also called Longitudinal Wave. They involve particle displacement in the incident plane and in the direction of propagation.
- Shear Vertical wave: They have particle displacement in the incident plane and perpendicular (dotted line) to the propagation direction (continuous line).
- Shear Horizontal wave: They have particle displacement perpendicular to the plane of incidence and perpendicular (see the point in the middle of the line) to the direction of propagation.

Guided waves include surface (Rayleigh) waves, which propagate along the free surface of a solid and concentrate their energy within roughly one wavelength of the surface.

## 5. Application Selection

Another aspect for selecting the wave mode according to the application is the angle of the transducer emission beam, which may be normal to the surface or oblique.

We can summarize the types of application according to the type of wave and its angle as follows in next subsections. It can be found in [1].

### 5.1. Applications for Bulk Waves

If bulk waves are emitted at a normal angle, whether they are longitudinal or transverse waves, they can be used for thickness measurement, crack detection, velocity measurement or measurement of material properties. However, if the bulk waves are emitted at an angle less than 90 degrees to the surface, whether they are SV or SH waves, they can be used for crack detection.

### 5.2. Applications of Guided Waves

Rayleigh surface waves are well suited to detect surface and near-surface defects, since their energy is confined to a thin layer below the surface.

### 5.3. Applications for Phase Array

**Figure 4:**
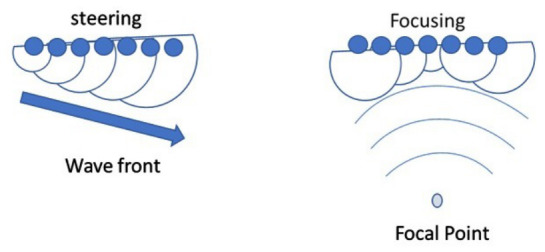
Ultrasound phased array.

Because EMAT coils are composed of several turns, each turn contributes to the sum of the magnetic field at a given point in the surrounding space. Thus, by having several turns of a coil distributed in space, depending on the distance between them and the direction of the current of each one, different forms of radiation pattern can be obtained. In Figure 4 we can observe two main effects [10]. At the left side, a steering effect can be observed, meanwhile at the right side, coil turns generates a focusing effect. These effects of phase array of spires are applied commonly with meander-line coils and other similar shapes, they will be described below in meander-line coils section.

Combining the wave-mode analysis above with the radiation patterns measured in Section 8 leads to the following coil-selection recommendation. Spiral coils (CSPCB, CS3D) generate shear vertical bulk waves with a broad main lobe oriented perpendicular to the specimen surface; this geometry is suitable for thickness measurement on plates and pipe walls. Meander-line coils (CMPCB, CM3D) concentrate the energy at an oblique angle set by the inter-turn spacing and the excitation frequency, favoring the generation of Rayleigh surface waves and therefore the detection of surface cracks. When a single probe must support both bulk and surface inspection, a meander-line coil with variable inter-turn spacing (LF-EMAT) provides a compromise, since it produces an oblique main lobe together with a secondary lobe that grazes the surface.

## 6. EMAT Coils Design

With the basics of ultrasound covered above, the next step is to study the different EMAT coil types and their effects on materials through their radiation patterns. The first criterion to classify an EMAT coil is whether it operates by Lorentz force or by magnetostriction.

**Figure 5:**
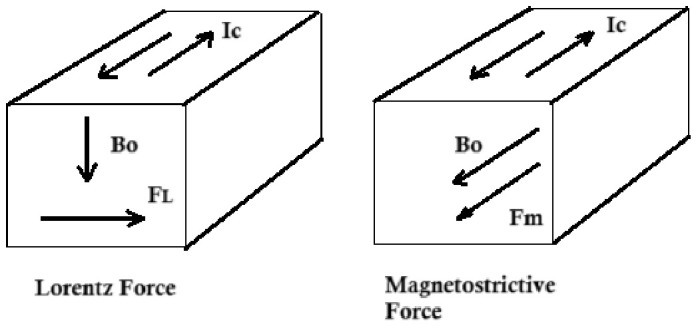
Predominant excitation mechanism in EMAT coils as a function of the geometric arrangement between the coil current $I_{c}$ and the bias magnetic field $B_{0}$. (**Left**): $I_{c}⊥B_{0}$ produces a Lorentz-force regime with particle motion perpendicular to $B_{0}$. (**Right**): $I_{c}\;\|\;B_{0}$ produces a magnetostriction regime in ferromagnetic materials.

The Figure 5 shows how a predominant Lorentz force effect is generated and, on the other hand, how the effect is generated with a predominant magnetostriction force [2]. On the left side of the figure, the coil currents (Ic) are perpendicular to the magnetic field (Bo), which generates a predominance of the Lorentz Forces.

On the other hand, on the right side of the Figure 5 it can be observed that the magnetic field (Bo) and the coil currents are parallel, generating a predominant effect of the magnetostriction force.

Lorentz-force EMAT coils are commonly applied to non-ferromagnetic conductors such as aluminum and austenitic stainless steels [11]. They are also applicable to ferromagnetic carbon steels when the bias magnetic field drives the material close to magnetic saturation: in that regime the magnetostrictive terms cease to contribute significantly to ultrasound generation, and the Lorentz force becomes the dominant excitation mechanism [12,13]. In the present work, the bias field is provided by a single grade-N52 NdFeB permanent magnet placed in close proximity to the steel specimen, which keeps the Lorentz mechanism dominant for the materials and geometries tested here.

### 6.1. Spiral Coils

**Figure 6:**
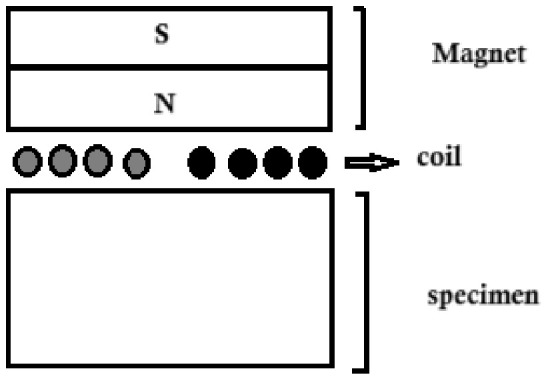
Arrangement of an EMAT spiral coil.

Spiral coils have the predominant effect of the Lorentz Force when the magnetic field is passed perpendicularly over them, which is achieved by placing a permanent magnet on the coils so that one of the poles points downwards. Figure 6 shows the arrangement of components of an EMAT with spiral coil based on Lorentz Force phenomena. This arrangement generates SH waves that propagate downwards. Thus, spiral coils are useful to thickness measures because ultrasonic waves are reflected on the back side of the specimen and echoes can be detected by the same coil that generates the waves.

**Figure 7:**
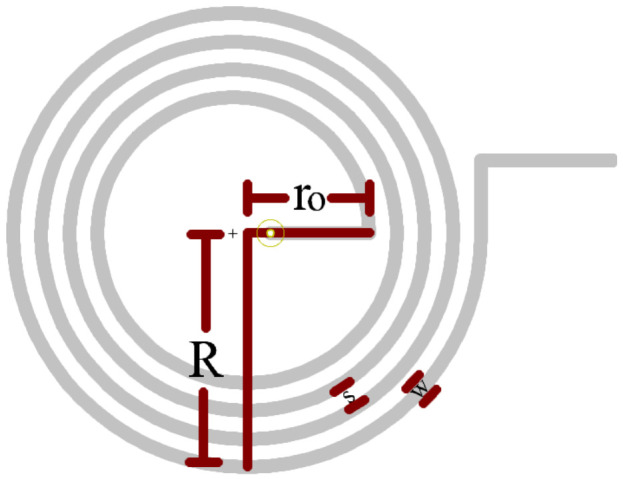
Geometrical parameters of an EMAT spiral coil.

Spiral coils operate within a range of working frequencies. The lowest frequency ($f_{L}$) is determined by the outer radius (*R*), while the highest frequency ($f_{H}$) is set by the inner radius ($r_{0}$). Figure 7 shows a basic spiral coil with its main parameters.

The frequency limits are calculated in [5] from (1) and (2), where *c* is the speed of sound in the material:

(1) $$f_{L}=\frac{c}{2\pi R},$$

(2) $$f_{H}=\frac{c}{2\pi r_{0}},$$

According to [5], the Archimedean spiral satisfies a linear dependence on the angle ϕ, expressed as:

(3) $$r_{1}=a\phi +r_{0},$$

(4) $$r_{2}=a\left(\phi -\pi \right)+r_{0},$$

where the growth rate *a* is defined by the arm width *w* and spacing *s*. For the self-complementary case (w=s), *a* reduces to:

(5) $$a=\frac{\left(w+s\right)}{\pi }=\frac{2w}{\pi }.$$

In such structures, when the width *w* and the spacing *s* are equal, the width of each arm is defined by:

(6) $$w=s=\frac{\left(R-r_{0}\right)}{2N}$$

with *N* the number of turns.

As mentioned in [3,14], for a spiral coil with the same radius, the magnetic field increases as the number of turns increases, and it can be modeled by Equation (7), with the tangential magnetic field $H_{x}^{V}$ at the surface of a material due to a unidirectional coil, with *n* turns per unit length and current *I*, where μ is the normalized permeability of the material.

(7) $$H_{x}^{V}\left(x,0\right)=H_{x}^{M}\left(x,0\right)=\frac{nI}{\mu +1}.$$

**Figure 8:**
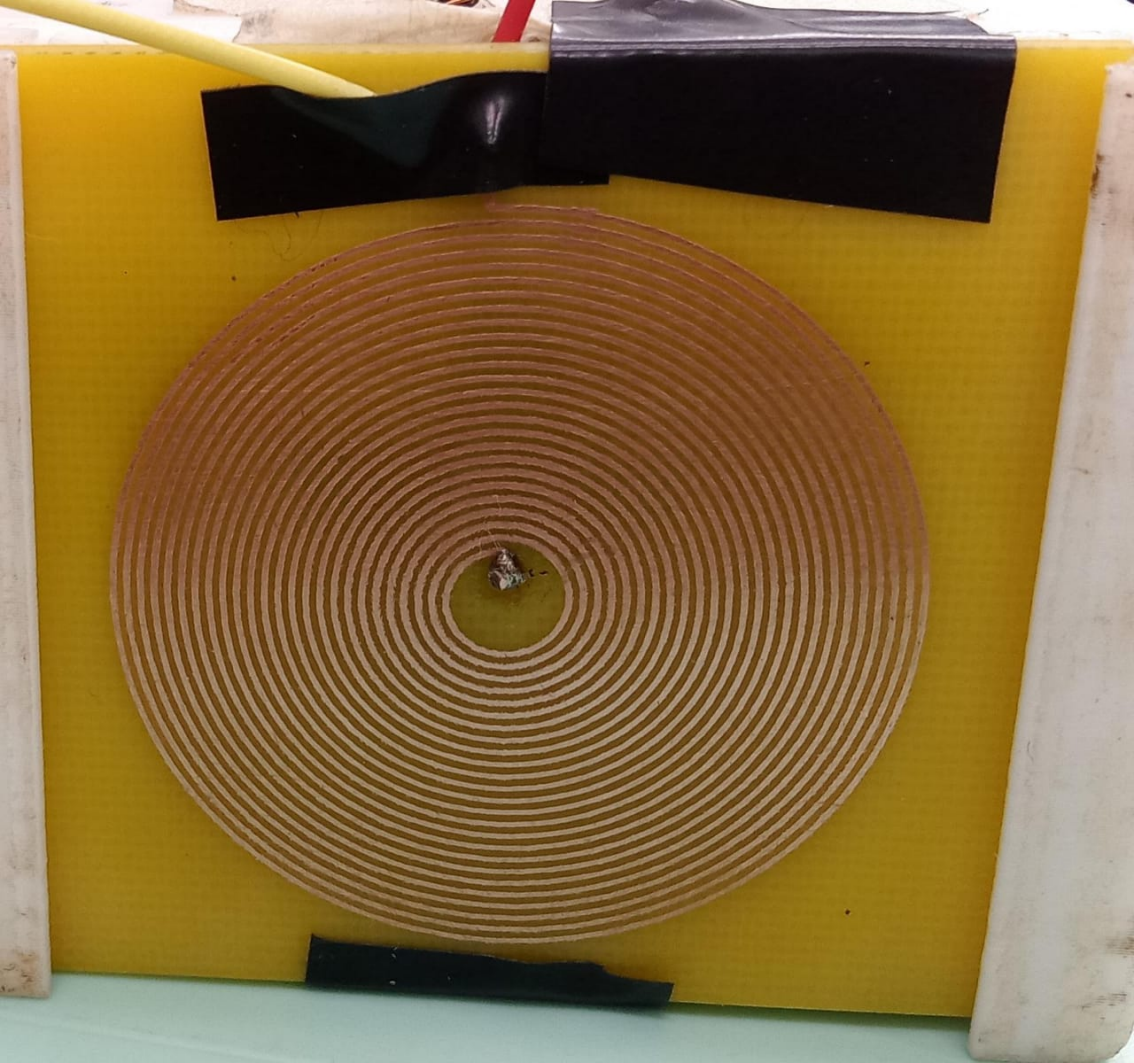
EMAT spiral coil fabricated on PCB (CSPCB).

In our work, a PCB coil design, referred to as CSPCB, was built and it is shown in Figure 8. This coil has 25 turns, R=20 mm and $r_{0}=2.5$ mm, with both the spacing and the track width set to 0.35 mm, according to the PCB design requirements. With these settings and a typical longitudinal sound velocity for steel of c=5900 m/s, the frequency bounds for this coil, computed from (1) and (2), are: $f_{L}=c/\left(2\pi R\right)\approx 46,950$ Hz and $f_{H}=c/\left(2\pi r_{0}\right)\approx 375,605$ Hz.

**Figure 9:**
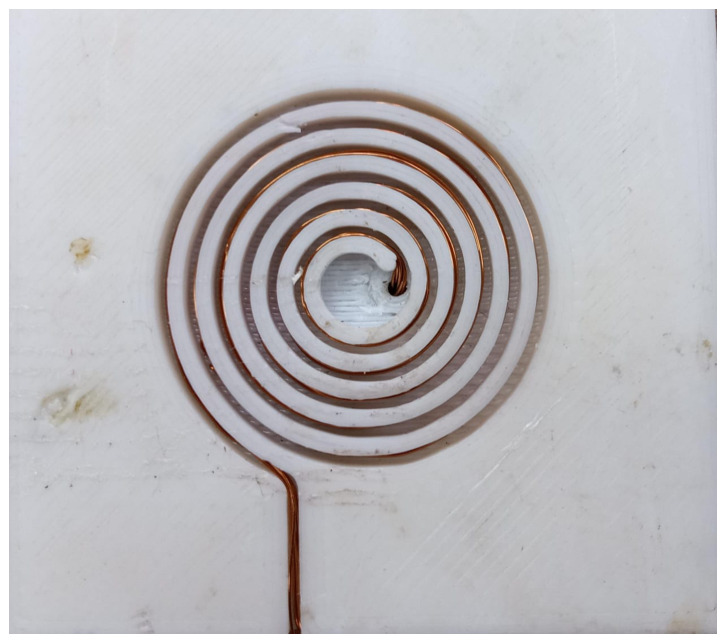
EMAT spiral coil fabricated using 3D-printing technology (CS3D).

A second spiral coil design, fabricated using 3D-printing technology and referred to as CS3D, is shown in Figure 9. This coil consists of 7 turns, with multiple wires passed through the printed channels, thereby forming a multi-turn spiral. The outer radius *R* is 15 mm and the inner radius $r_{0}$ is 1 mm, with both the spacing between turns and the track width set to 1 mm, according to the 3D-printing design requirements. Therefore, the limiting frequencies for this coils are: $f_{L}=62,600$ Hz and $f_{H}=939,014$ Hz.

In the section that describes the experiments with EMAT coils, radiation patterns of the CSPCB and CS3D spiral coils were obtained at the frequencies matching the simulations of Section 6.

### 6.2. Meander-Line Coil

Based on the articles [2,7,15,16], the behavior of this type of coil varies depending on the design configuration. In the prototype with a single coil, there are two subgroups: one with a fixed separation distance and another with variable distances, and the characteristics of each of these coil types are described below.

#### 6.2.1. Meander-Line Coil with Fixed Separation Distance

**Figure 10:**
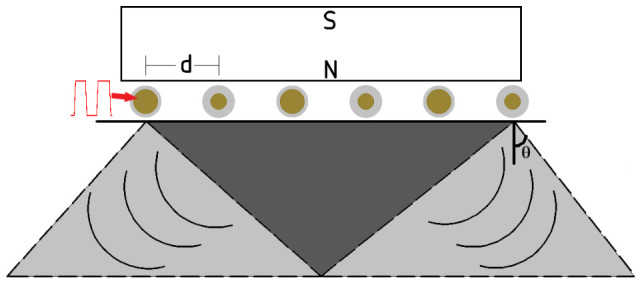
Schematic representation of a meander-line coil with constant inter-turn distance.

In this type of coil, it is important to maintain a constant separation distance between its paths, since, as shown in Figure 10, the wave propagates uniformly in two directions that are opposite to each other. This behavior can be described by Equation (8), which yields the maximum radiation angle for this configuration and relates the variables to be considered in its design.

(8) $$\sin\theta =\frac{c}{2df}=\frac{\lambda }{2d}$$

where

- *c* is the wave velocity.
- λ is the wavelength.
- *d* is the separation distance between paths.
- *f* is the excitation frequency.

**Figure 11:**
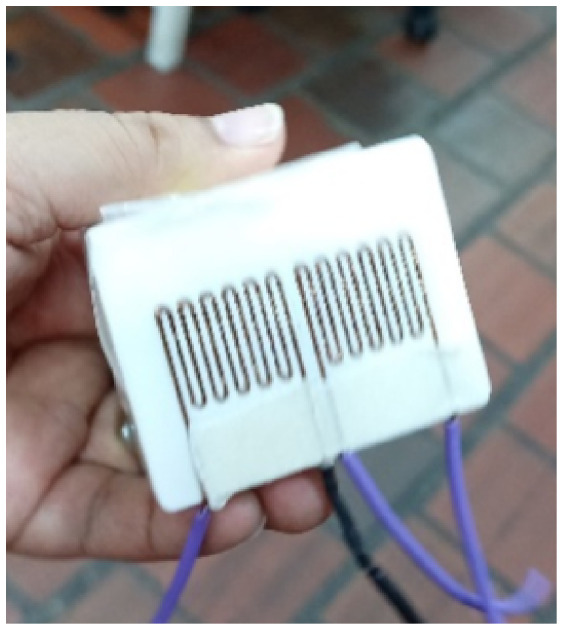
Meander-line EMAT coil built with a 3D printer (CM3D).

In this work, a meander-line coil with constant distance between turns was designed and built with a 3D printer (named CM3D) and with a distance of d=1 mm (see Figure 11). The resulting radiation pattern is shown in Figure 10, where it can be observed that the outgoing wave propagates in two opposite directions, which is consistent with the theoretical prediction.

#### 6.2.2. Meander-Line Coil with Variable Distance

The propagation direction of these waves depends on the progressive increase in the spacing between consecutive points of the coil. This variation enables the formation of a focal point that can be used for beam steering. Conversely, in the opposite direction, the outgoing wave propagates across a wider region of the specimen.

**Figure 12:**
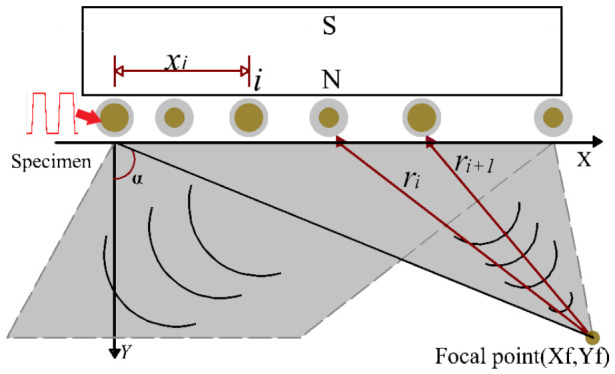
Direction of propagation with variable distance in a meander-line coil.

Figure 12 shows the schematic of a meander-line coil with variable spacing. In this configuration, a focal line is defined by Equations (9) and (10). The focal point is denoted as $\left(X_{f},Y_{f}\right)$ and is located at a steering angle α with respect to the origin. The distances from the focal point to the *i*-th and (i+1)-th elements of the coil are represented by $r_{i}$ and $r_{i+1}$, respectively. The coordinate $x_{i}$ corresponds to the position of the *i*-th coil element along the *x*-axis, while $y_{f}$ represents the *y*-coordinate of the focal point. It can be observed that the meander-line coil presents differences between the positive and negative paths, which are represented by the thickness of the elements located between the magnet and the specimen.

(9) $$r_{i}-r_{i+1}=\frac{c}{4\cdot f}=\frac{\lambda }{4}$$

(10) $$x_{i}=x_{f}-\sqrt{r_{i}^{2}-y_{f}^{2}}$$

Here, λ is the wavelength, *c* is the wave propagation velocity, and *f* is the frequency. The variables $r_{i}$, $r_{i+1}$, $x_{i}$, $y_{f}$, and α are illustrated in Figure 12 for clarity.

Experimental and numerical studies [16], supported by further experimental validation on defect detection [7], have demonstrated that the optimal steering angle for meander-line coils is approximately α = 30°, since this angle maximizes the SV-wave amplitude and improves focusing performance.

**Figure 13:**
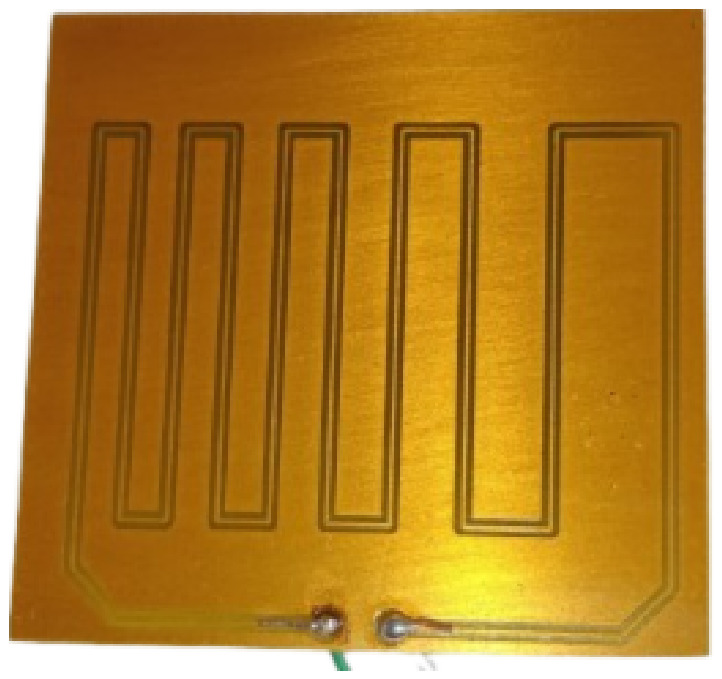
Meander-line coil PCB (CMPCB).

In this work, the coil was designed using the following parameters: $X_{f}=57.74$ mm, $Y_{f}=100$ mm, α = 30°, and f=500 kHz, as shown in Figure 13. The meander-line coil implemented in PCB design is referred to as CMPCB.

## 7. Simulation of EMAT Coil Radiation Pattern

EMAT coils can be viewed as antennas that emit waves in different directions within a material. Due to phenomena such as the Lorentz force and magnetostriction, changes in the magnetic field generate mechanical vibrations that propagate in different directions. These directions depend largely on the EMAT arrangement formed by the magnet and the coil, and in particular, the directions that the waves take depend on the geometric shape of the coil. Therefore, the shape of the EMAT coil is an important feature, and the direction in which it emits the waves will allow for one or more applications in the inspection of materials. In consequence, it is important to study the radiation patterns of the coils, and for this purpose, in this work simulations are used in the Comsol software to determine the shape of the radiation pattern.

The COMSOL Multiphysics model is configured as follows. **Material parameters** (steel specimen): relative permeability $\mu_{r}=1$, electrical conductivity $\sigma =4.032\times 10^{6}$ S/m and mass density ρ=7850 kg/m^3^; the coil conductor is modeled as copper with $\sigma_{Cu}=5.998\times 10^{7}$ S/m. **Excitation**: a static bias magnetic field produced by a grade-N52 NdFeB permanent magnet (surface remanence $B_{r}\approx 1.45$ T, coercivity $H_{c}\approx 52$ kA/m) applied normal to the coil plane, identical to the magnet used in the experiments, and an AC coil current I=128 A peak with a sinusoidal waveform at each tested frequency (500 kHz, 1.9 MHz and 4 MHz). **Boundary conditions**: a perfectly matched layer (PML) is applied at the outer boundary of the acoustic domain to absorb outgoing waves; the bottom face of the specimen is mechanically fixed; the lateral faces use low-reflecting boundary conditions; the magnetic problem uses magnetic insulation on the outer boundary. **Mesh**: free tetrahedral meshing with a global maximum element size of 0.4 mm and a refined region around the coil and the semicircular evaluation path with a maximum element size of 0.01 mm. The format and scope of the simulation-parameter list above follows the example of [17], where a similar set of material, excitation, mesh and solver parameters is used to describe a butterfly-coil EMAT model.

### 7.1. Simulation of Meanderline Coils

**Figure 14:**
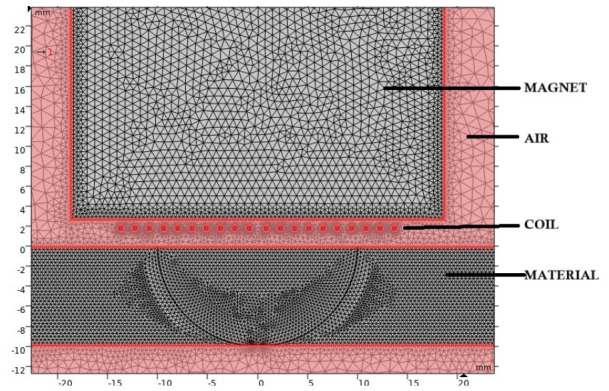
Framework for the simulation of meander-line EMAT coils.

For the simulations of this type of coil, two parameters were varied: distance between turns, and wave frequency. The arrangement of the simulation components is shown in the Figure 14. These components are: the magnet, the coil, and the material. As can be seen in the figure, the finite element simulation mesh changes for different materials, with smaller spacing at points near the edges and the von Mises stress measurement semicircle. Also, for meander-line coil, each turn has an opposite current direction with respect to its neighbors.

**Figure 15:**
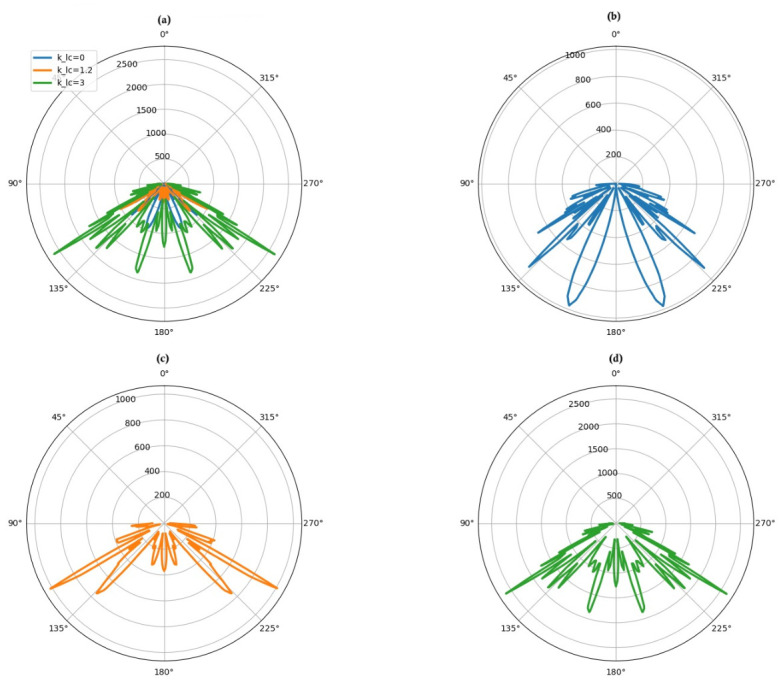
Radiation pattern of meander-line EMAT coils for several distances between turns.

To measure the radiation pattern, a semicircle was drawn along which the von Misses stress was measured. The values of this variable were then recorded along the semicircle and plotted on polar coordinates. The result is shown for a given frequency of 1.9 MHz, a modulation index (alpha factor) of 5, and several values for $k_{lc}$, which is an index that gives the number of times that distance between turns is over the diameter of the wire. Radiation pattern for several values of $k_{lc}$ parameter is shown in the Figure 15. That figure has four images: (a) is a comparison of amplitudes at three distances between turns, (b) is the radiation pattern at 0 distance between turns, (c) is the radiation pattern for a distance of 1.2 diameters, and (d) is the radiation pattern for a distance of 3 diameters between turns. As shown in the figure, for a constant frequency, the angle of the main lobes varies with the distance between turns. This behavior is consistent with the equation for angle calculation in meander-line coils, as presented in the coil design section. Furthermore, it can be observed that when the distance between turns is zero, two distinct main lobes are obtained. In contrast, for a distance of 1.2 diameters, four main lobes are observed, while for a distance of 3 diameters, multiple main lobes appear at different angles, accompanied by an increase in amplitude.

Figure 15 exposes two design trade-offs for meander-line coils. First, when the inter-turn separation grows while the outer coil dimensions remain fixed, the number of active turns inside the coil footprint decreases; the per-turn current density therefore rises and the peak amplitude of the main lobe increases. Second, the same wider spacing distributes the constructive interference over a broader angular range, which lowers the directivity of the main lobe and raises the number and amplitude of secondary lobes. The inter-turn spacing must therefore balance signal strength against angular selectivity according to the target application.

**Figure 16:**
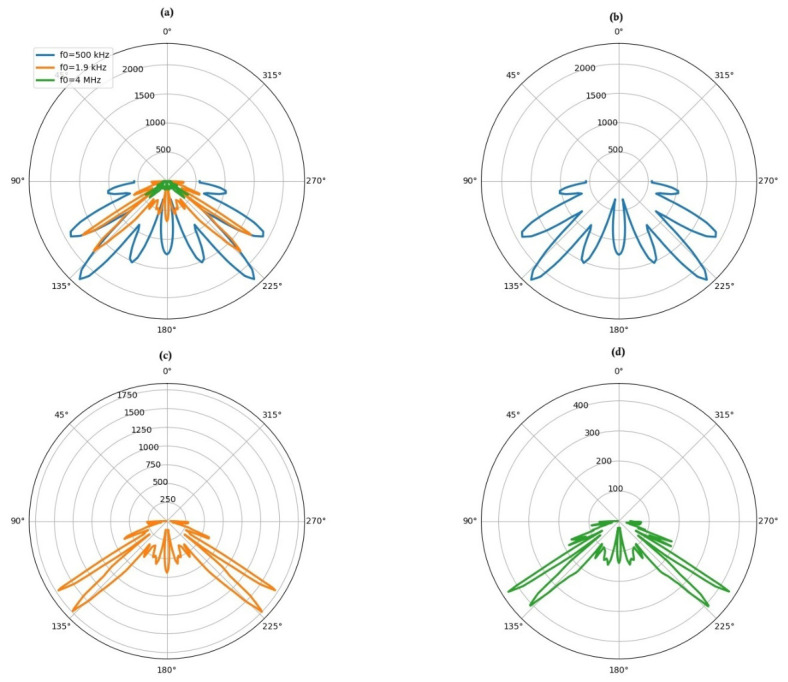
Radiation pattern of meander-line EMAT coils for several frequencies.

Another important issue is the behavior of radiation pattern versus the wave frequency. In this study, frequency values were: 500 kHz, 1.9 MHz and 4 MHz. Radiation patterns for those values are shown in Figure 16. In that simulation, index $k_{lc}$ for distance between turns is 1.5 and modulation index (alpha factor) is 5. Also, in (a) part of the figure, a comparison between radiation patterns at each value of frequency is showed. An important conclusion is that at 500 kHz, amplitude is higher and in (b) part of that figure, the lobes are wider than at higher frequencies (parts c and d). Another aspect is that main lobes change their angle with the frequency.

**Figure 17:**
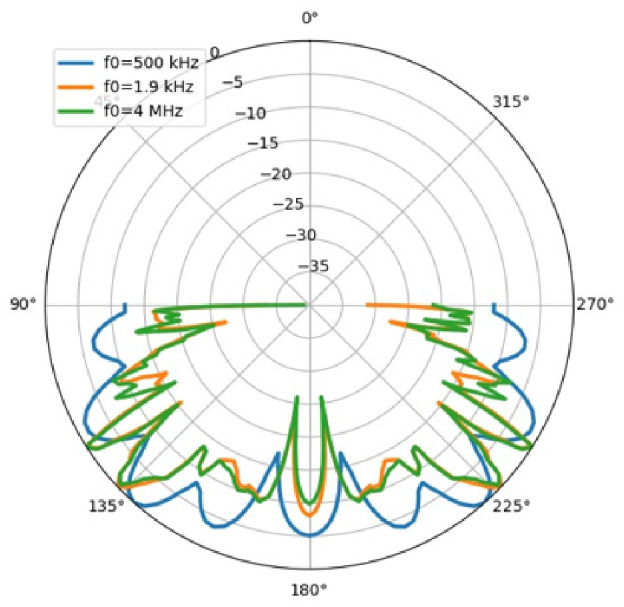
Radiation pattern (dB) of meander-line EMAT coils for several frequencies.

To visualize the radiation patterns in a manner comparable to actual measurements, the patterns were expressed in decibels (dB). Due to space constraints, only the dB radiation patterns corresponding to frequency variations are presented in Figure 17. It can be observed that the radiation patterns in dB exhibit two principal lobes at frequencies of 1.9 MHz and 4 MHz. At 500 kHz, however, the main lobes are not clearly defined. Furthermore, it is important to note the presence of smaller lobes oriented toward superficial directions. These lobes are associated with Rayleigh waves and they can be utilized for the detection of cracks on material surfaces.

### 7.2. Simulation of Spiral Coils

For spiral coils, the simulation framework is identical to that presented in Figure 14, and the finite element mesh remains unchanged. The distinction arises from the current distribution in each turn. Specifically, in a spiral coil, all conductors located to the left of the coil’s center carry current in one direction, whereas those positioned to the right of the center carry current in the opposite direction.

**Figure 18:**
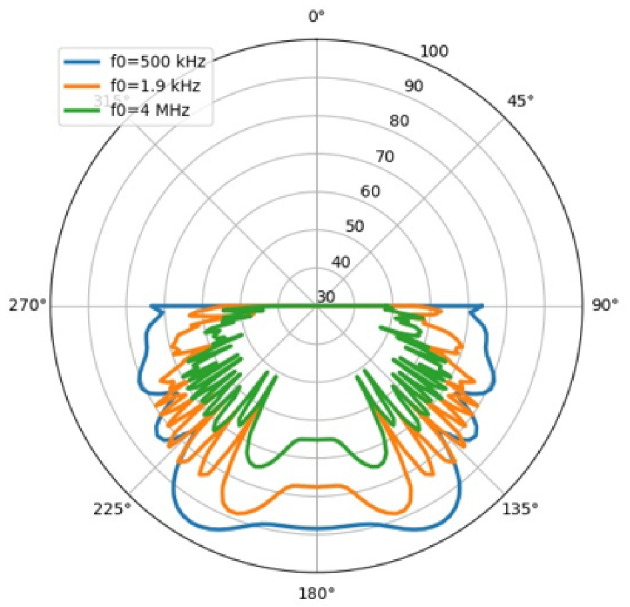
Radiation pattern of EMAT spiral coils for several frequencies.

For the spiral coil simulations, the following parameters were varied: signal frequency, and distance between coils. In Figure 18 radiation pattern is simulated for three frequency values: 500 kHz, 1.9 MHz and 4 MHz. The figure shows in all frequencies a large, almost triangular-shaped lobe, which points perpendicularly to the surface of the material. This makes this type of coil primarily usable in thickness measurement applications.

### 7.3. Simulation of LF EMAT Coils

**Figure 19:**
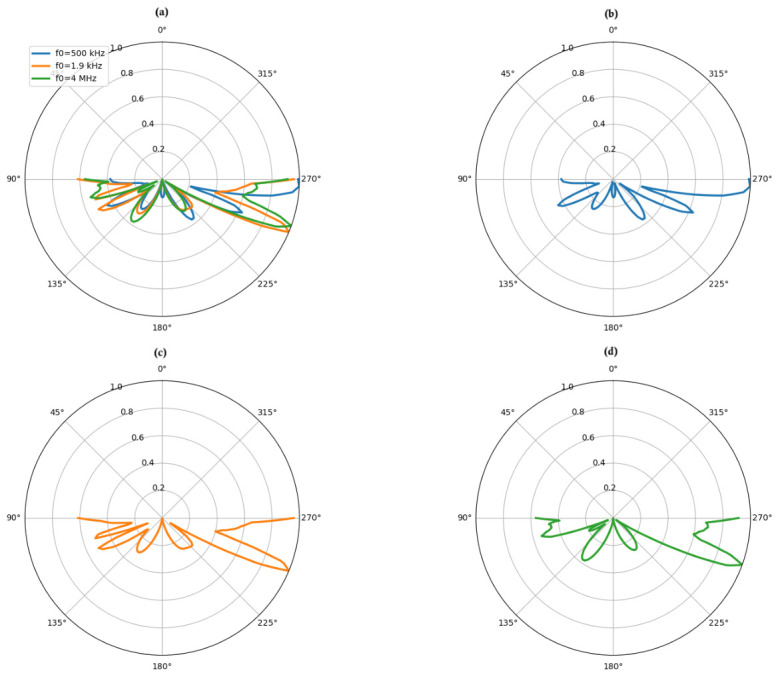
Radiation pattern of the LF-EMAT coil for several frequencies.

LF EMAT coils (Line Focus EMAT) consist of an array of coils similar in shape to meander coils, but with variable distances between them. This allows this type of coil to focus the beam at a point within the material at a specific angle relative to the material’s surface. This behavior can be observed in Figure 12. Simulation results for frequency variation can be observed in Figure 19. It can be observed that there is one main lobe pointing to the right side in all cases. However, the angle of the highest amplitude differs. In case (b), the frequency is 500 kHz, and the main lobe points almost toward the surface side of the material. In case (c), the frequency is 1.9 MHz, and the angle is close to 22 degrees from the surface. In case (d), the frequency is 4 MHz, and the angle is almost the same as in the 1.9 MHz case. In all cases, there is a small lobe pointing to the left side, which generates a wave that travels along the surface (Rayleigh wave). In case (a), when all cases are compared, it is clear that the amplitude of the main lobes is similar.

## 8. Experiments with EMAT Coils

**Figure 20:**
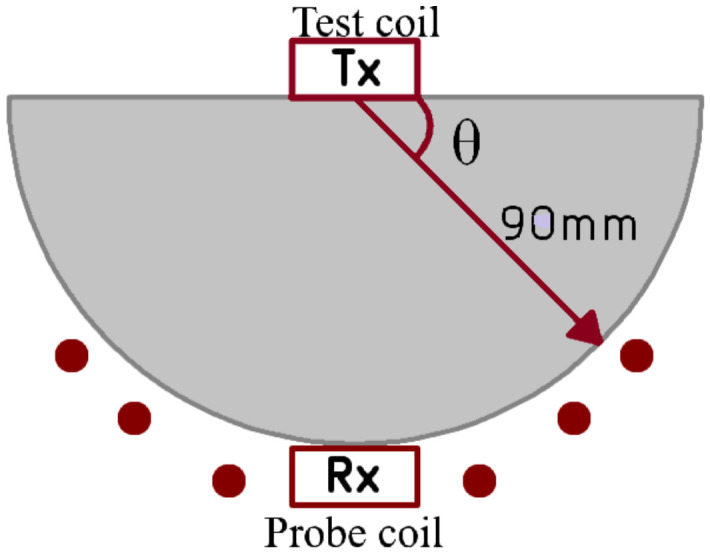
Experimental setup for the radiation-pattern measurement of EMAT coils. The radius between the transmitter (Tx) and the receiver (Rx) coil is 90 mm.

Based on the antenna theory presented in [18], where the methodology for measuring radiation patterns is defined, an analogous approach can be applied to coils, as illustrated in Figure 20. In this setup, two coils are employed: the test coil (Tx), which generates the field to be characterized and is placed on the flat plane of the setup, and the probe coil (Rx), which receives the signal amplitude while rotating across different angles. The probe coil was the same for all measurements and consisted of a spiral coil with 100 turns.

In this work, the measurement is performed over a half-moon arc ranging from 10° to 170°, excluding the extremes at 0° and 180°. These angles are avoided because at these positions the probe coil does not establish proper coupling with the test coil, which may lead to inaccurate results.

The experimental setup used for this purpose is shown in Figure 20. Each marked position represents one of the 16 measurement points at which the signal amplitude was recorded. The distance between the coils is 90 mm in radius. In all coils, the process starts from the lowest angle and progresses to the maximum angle for the subsequent representations of the radiation pattern.

The bias magnetic field at the test coil is generated by a single grade-N52 NdFeB permanent magnet placed on the back of the coil, providing a surface remanence $B_{r}\approx 1.45$ T and a coercivity $H_{c}\approx 52$ kA/m. The same magnet is used both in the experimental measurements and in the COMSOL simulations of Section 7, so that the bias field condition is identical in both campaigns. With this excitation, the Lorentz mechanism dominates the wave generation for the steel specimens used in the experiments.

## 9. Results and Discussion

In order to provide a direct comparison between simulation and experiment, the experimental campaign was repeated at the same frequencies used in the COMSOL simulations of Section 6 (500 kHz, 1.9 MHz and 4 MHz) on the same steel specimen and with the same set of fabricated coils. The polar plots presented in this section therefore correspond exactly to the simulated cases, which strengthens the validity of the model verification. The maximum amplitudes reported below and summarized in Table 2 are the raw peak voltages observed in the measurements.

### 9.1. Spiral Coils

Radiation patterns for spiral coils were measured at 500 kHz, 1.9 MHz and 4 MHz, matching the frequencies used in the COMSOL simulations.

**Figure 21:**
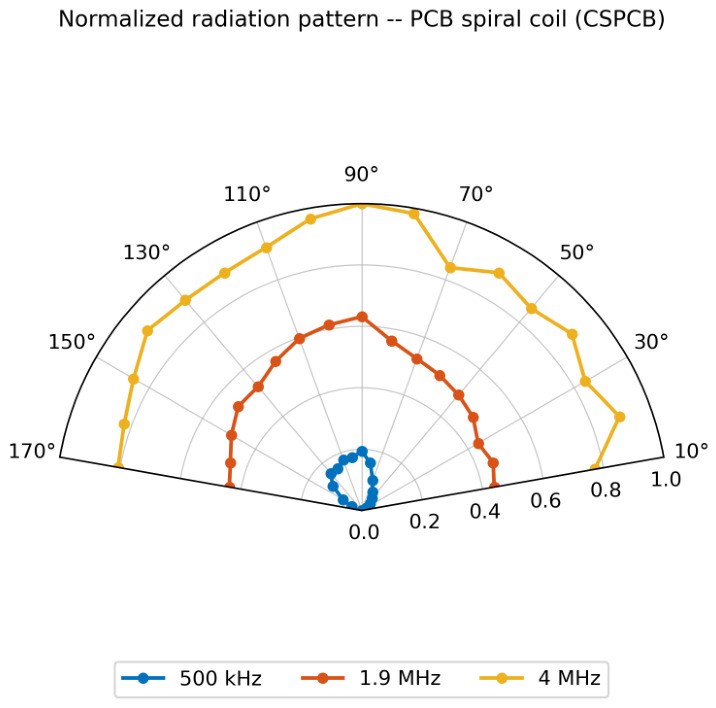
Normalized radiation pattern of the PCB spiral coil (CSPCB) measured at the simulation frequencies (500 kHz, 1.9 MHz, 4 MHz).

For the PCB spiral coil, as shown in Figure 21, the radiation pattern covers a wide angular range of the specimen. The maximum amplitude was 37.3 mV at 4 MHz, with 32.7 mV at 500 kHz and 35.2 mV at 1.9 MHz; for this coil the amplitude grows monotonically with frequency in the tested range.

**Figure 22:**
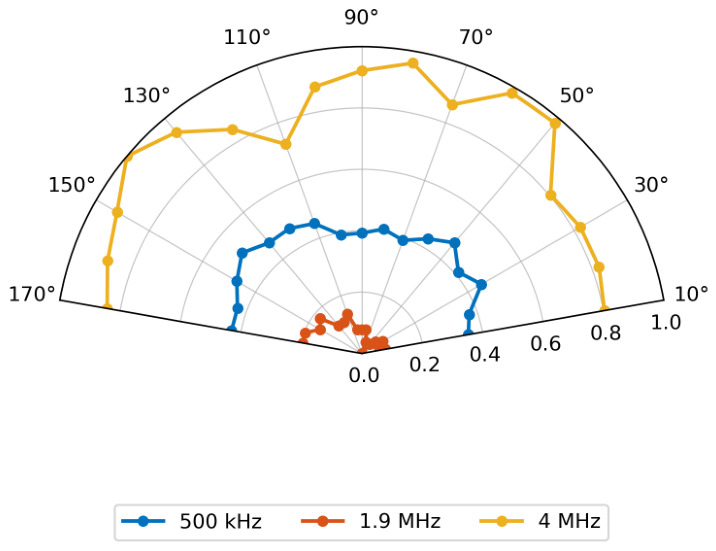
Normalized radiation pattern of the 3D-printed spiral coil (CS3D) measured at the simulation frequencies (500 kHz, 1.9 MHz, 4 MHz).

For the 3D-printed spiral coil, as shown in Figure 22, the radiation pattern also covers a wide angular range of the specimen. The maximum amplitude was 39.6 mV at 4 MHz, with 37.1 mV at 500 kHz and 35.5 mV at 1.9 MHz. In the range from 110° to 170° the amplitude decreases slightly. The simulated and measured radiation patterns of the spiral coils share their main feature: the largest signal is obtained along the direction perpendicular to the specimen surface for all tested frequencies. The simulated lobe is sharply triangular, while the measured one is smoother and crescent-shaped because of the broader angular sensitivity of the receiver coil and the finite spatial resolution of the bench; in both cases the peak position and the perpendicular gain match.

### 9.2. Meander-Line Coils

In the case of meander-line coils, the behavior depends on the separation of the construction paths and the wave frequency.

**Figure 23:**
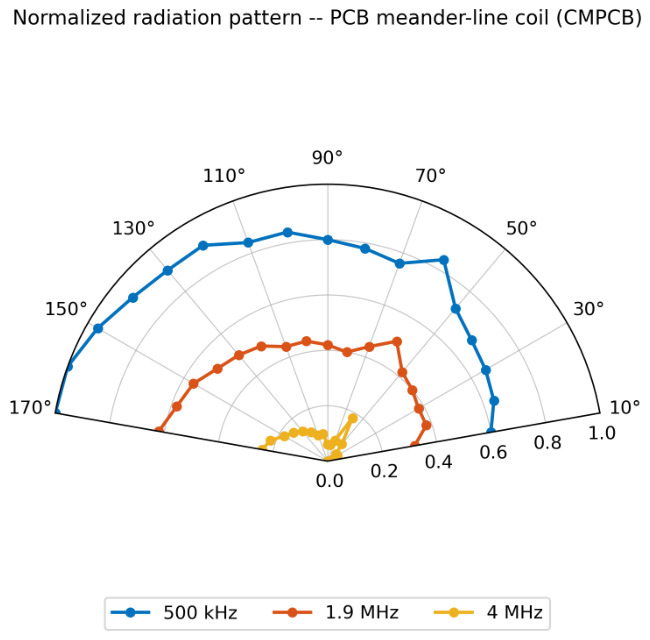
Normalized radiation pattern of the PCB meander-line coil (CMPCB) measured at the simulation frequencies (500 kHz, 1.9 MHz, 4 MHz).

For the PCB meander-line coil with variable distance, also named LF EMAT, as shown in Figure 23, the radiation pattern shows a peak pointing at an oblique angle to the right side. At the simulation-matched frequencies, the maximum amplitude is 23.4 mV at 500 kHz, 21.5 mV at 1.9 MHz and 19.6 mV at 4 MHz. This coil also exhibits a secondary lobe to the left side, grazing the specimen surface, which is useful for Rayleigh wave generation. The peak angle is approximately 30° from the vertical axis (about 60° with respect to the surface) and remains stable across the tested frequencies, in agreement with the theoretical prediction.

**Figure 24:**
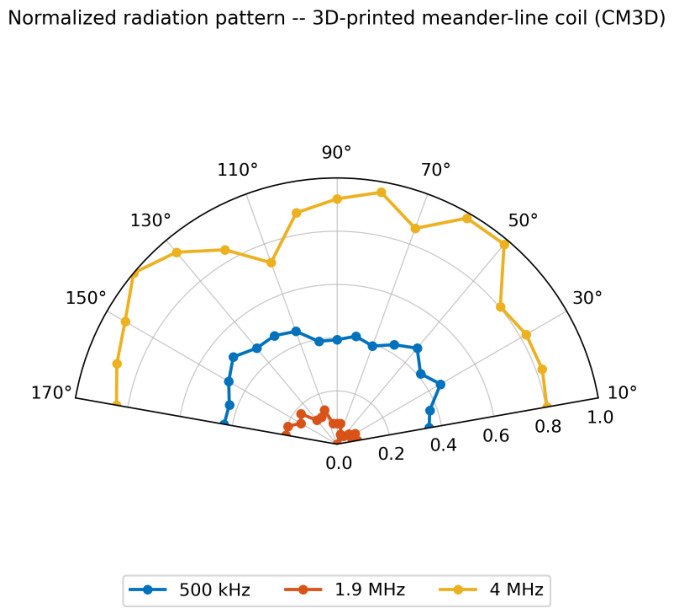
Normalized radiation pattern of the 3D-printed meander-line coil (CM3D) measured at the simulation frequencies (500 kHz, 1.9 MHz, 4 MHz).

For the 3D-printed meander-line coil (CM3D), the measured radiation pattern at the simulation frequencies is shown in Figure 24. The maximum amplitude is 39.6 mV at 4 MHz, with 37.1 mV at 500 kHz and 35.5 mV at 1.9 MHz. The pattern has approximately three main lobes at 4 Mhz (at 30, 80 and 140 degrees), but at 500 Khz the pattern points to 30 and 140 degrees along the explored angular range.

### 9.3. Summary of Results

As summarized in Table 2, the maximum measured amplitudes at the simulation-matched frequencies are 37.3 mV for the PCB spiral coil (CSPCB) at 4 MHz, 39.6 mV for the 3D-printed spiral coil (CS3D) at 4 MHz, 23.4 mV for the PCB meander-line coil (CMPCB) at 500 kHz and 39.6 mV for the 3D-printed meander-line coil (CM3D) at 4 MHz.

Spiral coils yield higher amplitudes than meander-line coils due to their larger number of turns, which makes them suitable when a higher output amplitude is required. Both spiral models tested (CSPCB and CS3D) also exhibit stable behavior across frequency, covering a wide angular range, which makes them suitable for thickness measurements. Meander-line coils, with fewer turns, produce lower amplitudes and show greater variability with frequency; the measurements nevertheless agree with the theoretical prediction, which makes these coils suitable when a specific output angle is required and for Rayleigh-wave generation aimed at surface-crack detection.

## 10. Conclusions

This paper presents a methodology for the design of EMAT coils based on the Lorentz force. All steps of the methodology are described and applied: theoretical concepts, application selection, radiation-pattern simulation, fabrication and experimental validation. While other works study spiral or meander-line coils separately, few works compile the design of both types together with the underlying ultrasound foundations in a single document.

A further contribution is the analogy drawn with radio-frequency antennas to study the behavior of EMAT coils, using concepts such as a transmitting coil, a receiving coil and the radiation pattern. This concept is underused in the EMAT literature and aids the interpretation of the coil response.

Additive manufacturing (3D printing) was also used as a coil-fabrication technology, alongside conventional PCB technology.

The paper also introduces ultrasound concepts that apply to both conventional ultrasound and EMAT-generated ultrasound, since ultrasound waves behave the same way in a material regardless of the excitation mechanism. The basics of EMAT generation are then reviewed for the two coil families used in this work, those operating by Lorentz force and those operating by magnetostriction; the dominant mechanism is set by the geometric arrangement between the magnet and the coil. The design equations for spiral and meander-line coils are derived, simulations are performed for both topologies, and laboratory measurements of the radiation pattern are reported for coils built with PCB and 3D-printing technologies.

All radiation-pattern measurements reported in this work used the same input excitation: a rectangular wave of 10 V peak amplitude. The attenuation between the input excitation and the peak received voltage, $A_{\mathrm{dB}}=-20\log_{10}\left(V_{\max}/10\;V\right)$, yields −48.6 dB for CSPCB, −48.0 dB for CS3D and CM3D, and −52.6 dB for CMPCB. The largest signal-to-noise penalty, around 4–5 dB of extra attenuation, corresponds to the PCB meander-line coil (CMPCB), in agreement with its lower number of effective turns and the narrow main lobe that concentrates the energy off the receiver axis. The spiral coils, with a larger number of turns and a wide main lobe oriented perpendicular to the surface, yield the best SNR. The two parameters most adversely affecting SNR in the present experiments are therefore (i) a small effective number of turns and (ii) a strong angular directivity that concentrates the energy outside the receiver position.

For meander-line coils, increasing the spacing between turns raises the radiated amplitude but reduces directivity and introduces additional secondary lobes; this trade-off must be balanced against the application requirements.

## Figures and Tables

**Table 2:** Summary of the frequency at which the maximum amplitude is observed and the corresponding maximum amplitude for each coil, measured at the simulation-matched frequencies (500 kHz, 1.9 MHz, 4 MHz).

Reference	Coil	Frequency of Maximum Amplitude (kHz)	Maximum Amplitude (mV)
CSPCB	PCB spiral coil	4000	37.3
CMPCB	PCB LF EMAT coil	500	23.4
CS3D	3D spiral coil	4000	39.6
CM3D	3D meander-line EMAT coil	4000	39.6

## Data Availability

The datasets presented in this article are not readily available because the data are part of an ongoing study. Requests to access the datasets should be directed to Jhon Padilla, the correspondence author.

## Abbreviations

The following abbreviations are used in this manuscript:

EMAT	Electro Magnetic Acoustic Transducer
NDT	Non Destructive Testing
SV	Shear Vertical Wave
SH	Shear Horizontal Wave
PCB	Printed Circuit Board
3D	Three Dimensional
CSPCB	Coil Spiral Printed Circuit Board
CS3D	Coil Spiral 3D Printed
LF	Line Focus
CM3D	Coil Meander Line 3D Printed
CMPCB	Coil Meander Line Printed Circuit Board
LF EMAT	Line Focus Electro Magnetic Acoustic Transducer
